# Candidiasis profile at the outpatient department of the university of cape coast hospital in the central region of Ghana: a retrospective study

**DOI:** 10.1186/s12905-023-02253-y

**Published:** 2023-03-10

**Authors:** Kwame Kumi Asare, Hilda Amuaku Bentil, Emmanuel Gyesi, Samuel Amoah, Felicity Bentsi-Enchill, Yeboah Kwaku Opoku

**Affiliations:** 1 Department of Biomedical Science, School of Allied Health Sciences, College of Allied Health Sciences, University of Cape Coast, Cape Coast, Ghana; 2 Biomedical and Clinical Research Centre, College of Allied Health Sciences, University of Cape Coast, Cape Coast, Ghana; 3 Department of Laboratory, University of Cape Coast Hospital, Cape Coast, Ghana; 4 Department of Biology Education, Faculty of Science Education, University of Education, Winneba, Ghana

**Keywords:** Vulvovaginal candidiasis, Candida albicans, Pus cells (PCs), Red Blood cells (RBCs), Epithelial Cells (ECs), Diagnosis

## Abstract

**Introduction:**

Vulvovaginal candidiasis (VVC) is a public health problem with an estimated 138 million women globally experiencing recurrent VVC annually. The microscopic diagnosis of VVC has low sensitivity, but it remains an essential tool for diagnosis as the microbiological culture methods are limited to advanced clinical microbiology laboratories in developing countries. The study retrospectively analyzed the presence of red blood cells (RBCs), epithelial cells (ECs), pus cells (PCs) and *Candida albicans* positive in wet mount preparation of urine or high vaginal swabs (HVS) samples to test for their sensitivity and specificity for the diagnosis of candidiasis.

**Methods:**

The study is a retrospective analysis at the Outpatient Department of the University of Cape Coast between 2013 and 2020. All urine and high vagina swabs (HVS) cultures samples using Sabourauds dextrose agar with wet mount data were analyzed. 2 × 2 contingency diagnostic test was used to ascertain the diagnostic accuracy of red blood cells (RBCs), epithelial cells (ECs), pus cells (PCs), and *Candida albicans* positive in wet mount preparation of urine or high vaginal swabs (HVS) samples for the diagnosis of candidiasis. The association of candidiasis among patients' demographics was analyzed using relative risk (RR) analysis.

**Results:**

The high prevalence of candida infection was among female subjects 97.1% (831/856) compared to males 2.9% (25/856). The microscopic profiles which characterized candida infection were pus cells 96.4% (825/856), epithelial cells 98.7% (845/856), red blood cells (RBCs) 7.6% (65/856) and *Candida albicans* positive 63.2% (541/856). There was a lower risk of Candida infections among male patients compared to female patients RR (95% CI) = 0.061 (0.041–0.088). The sensitivity (95%) for detecting *Candida albicans* positive and red blood cells (0.62 (0.59–0.65)), *Candida albicans* positive and pus cells (0.75 (0.72–0.78)) and *Candida albicans* positive and epithelial cells (0.95 (0.92–0.96)) with corresponding specificity (95% CI) of 0.63 (0.60–0.67), 0.69 (0.66–0.72) and 0.74 (0.71–0.76) were detected among the high vaginal swab samples.

**Conclusion:**

In conclusion, the study has shown that the presence of PCs, ECs, RBCs or ratio of RBCs/ECs and RBCs/PCs in the wet mount preparation from urine or HVS can enhance microscopic diagnosis of VVC cases.

## Introduction

Vulvovaginal candidiasis (VVC) is a public health problem that affects millions of women globally [1, 2]. At least 75% of all women are estimated to experience vaginal candida infection at least once in their lifetime [3]. Also, approximately 50% suffer from recurrent infection. VVC is the second most common cause of vaginitis after bacterial infections [4]. VVC infections are either asymptomatic or symptomatic infections and an estimated one-third of all women exhibit no symptoms [5]. The vulvar itching and abnormal vaginal odourless ‘cheese-like’ or watery discharges characterize clinical symptomatic of candidiasis [6]. *Candida albicans* infection constitutes about 85–95% of all VVC and 5–10% of non-albicans infections such as *Candida glabrata* and *Candida krusei* [7, 8].

VVC morbidity is associated with pain, altered self-esteem, impairing work performance, discomfort, interfering with sexual and affective relations, mental distress and at considerable direct and indirect economic costs [7]. An estimated 138 million women globally experience recurrent VVC annually [9, 10]. The burden from lost productivity due to VVC in high-income countries could cost about US$14.39 billion annually by 2030 [10]. The economic losses, the morbidity and the increasing prevalence of recurrent VVC necessitate urgent solutions and improved quality of care for affected women [11, 12].

Effective diagnosis of VVC is essential for providing prompt and improved treatment against candida infections. Frequently, diagnosis of candida vaginitis depends on clinical symptoms with or without microscopic verification [13]. However, Candida vaginitis symptoms are not specific to candida infections and could result in misdiagnosis. Microscopic diagnosis of VVC has low sensitivity and almost 50% of culture-positive candidiasis from symptomatic patients showed false-negative in microscopic diagnosis [14, 15]. Although the microscopic diagnosis of candida infections has low sensitivity, it remains an essential tool for VVC diagnosis as it could exclude other causes of vaginitis [15].

Culture diagnosis remains the ‘gold standard’ method for Candida infections with high sensitivity and specificity compared to microscopic diagnosis [16]. However, the microbiological culture methods are limited to advanced clinical microbiology laboratories, especially in developing countries. Also, candida culture has a lag identification time of five days which delays prompt diagnosis [17]. The molecular diagnostic method is rapid, highly sensitive and specific for the detection of Candida species but requires a skillful technique with a high cost of implementation [18]. It is, therefore, typically reserved for advanced research laboratories in developing countries.

The study retrospectively analyzed the presence of red blood cells (RBCs), epithelial cells (ECs), pus cells (PCs) and *Candida albicans* positive in wet mount preparation of urine or high vaginal swabs (HVS) samples tested for their sensitivity and specificity for the diagnosis of candidiasis. The data excluded all mixed candida and other infections from the analysis. Diagnosed candidiasis by culture with wet mount data was included in the final analysis to assess diagnostic potential of candidiasis profiles in developing countries.

## Methods

### Study area

**Fig. 1:**
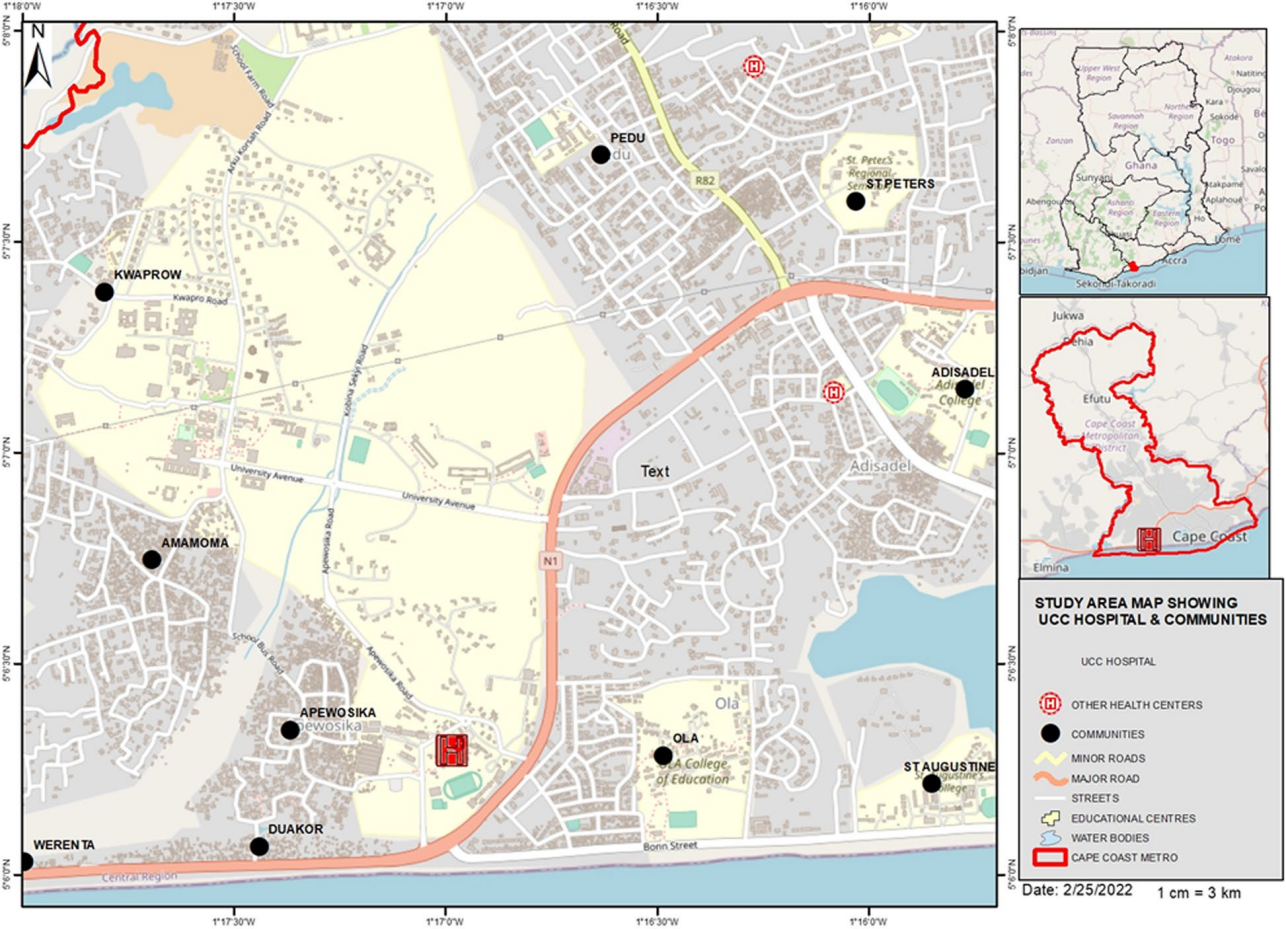
The map of the University of Cape Coast community

The University of Cape Coast Hospital is situated at the southern end of the University of Cape Coast (UCC) which is located in the Cape Coast Metropolis of the Central Region of Ghana. The hospital was established in 1962 and currently has a bed capacity of 75 with a staff capacity of 275. The hospital provides 34 healthcare services including pediatric services, radiology departments, diabetic clinics, hypertension clinics, obstetrics, gynecological services, ENT services, dental, laboratory services, and pharmaceutical services to the university community. The university is a sea-front university that lies between 50 8′ 10’’ N, 10 17′ 56’’ W to NE and 50 5′ 51’’ N, 10 16′ 43’’W to SE. It is located along the shores of the Gulf of Guinea, which spans the west coast of West Africa. The University hospital is about 160 m from the shores of the Gulf of Guinea and is located at the main entrance of the University campus. The University shares boundaries with Akotokyir, Kwaprow, Amamoma, Apewosika and Duakor communities. In a broader view, it is found within the Cape Coast Metropolitan areas which has Cape Coast city as its capital. The metropolis is bounded on the south by the Gulf of Guinea, west by the Komenda-Edina-Eguafo-Abirem (KEEA) Municipality, east by the Abura-Asebu-Kwamankese District and the north by the Twifo-Hemang Lower Denkyira District. The university hospital provides health services to students, University staff and the surrounding communities (Fig. 1).

### Data collection and eligibility criteria

**Fig. 2:**
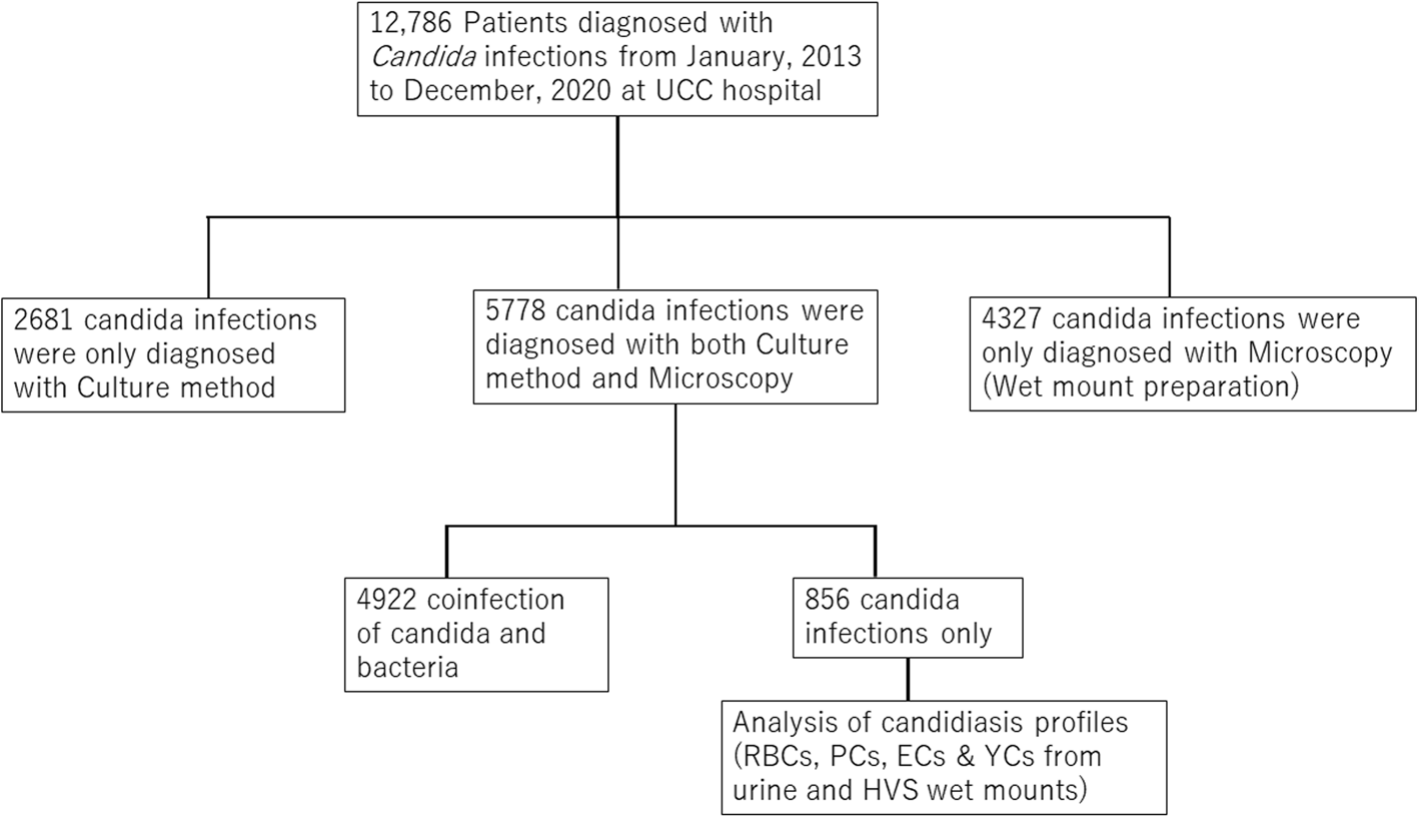
Flow chart showing the screening process of candidiasis diagnosis at the university of Cape Coast hospital for the retrospective analysis

The laboratory register for candida diagnosis data was collected and analysed from January 2013 to December 2020. A total of 12,786 patients who attended the Outpatients department of the hospital were diagnosed with candida infection within the period under review. Patients with Candida infections diagnosed based on only culture (2,681) and patients who were diagnosed with only microscopy (4,327) were excluded from the data analysis. The remaining 5778 patients who had both microscopic and culture diagnoses were further screened for only Candida infection. Out of the 5,778; 4,922 representing over 85% of the patients had co-infection of candida and bacterial infections and only 856 of the patients diagnosed with only candida infections were analyzed for candidiasis profiles (Fig. 2).

### Variables and definitions

Candidiasis profiles are defined as the characteristic appearance of the wet mount of candida samples under a high-power field light microscopy. The candidiasis profiles under consideration were epithelial cells (ECs), pus cells (PCs), red blood cells (RBCs) and *Candida albicans* positive in a wet mount preparation. The patients’ demographic characteristics including patient age, sex, the type of samples and the year of diagnosis were also obtained.

### Sample collection

The urine and HVS samples were collected and analyzed for candidiasis and candidiasis profiles from patients who visited the Outpatients department of the University of Cape Coast Hospital from January 2013 to December 2020. The patients who provided urine samples were given a screw-cap plastic container to collect 25 to 30 ml of midstream urine samples. For vaginal swabs, patients were instructed verbally on how to collect high vagina swabs (HVS) samples by asking them to insert the swab into the vagina approximately 4 to 5 cm and then rotate it several times before placing it into a capped tube. Again, they were given a diagram showing the procedure for HVS sample collection.

### Isolation of *Candida sp*.

The urine samples were centrifuged at 3000 rpm for 2 min and emulsified HVS samples in peptone water; 20 ul of the urine deposit or the emulsified HVS were spread on the SDA agar plate using a sterilized glass rod. Urine and the HVS samples were cultured on Sabourauds dextrose agar plates, containing 0.5 mg per 1000 ml chloramphenicol and incubated at 37 ^o^ C and examined for its growth at 24, 48 and 72 h. The culture plates were examined for the appearance, size, cream-coloured pastry colonies and morphology of the colonies [19].

### Microscopic identification of *Candida sp*. and candidiasis profile in a wet mount and culture

The wet mount preparation of urine and HVS sample were observed and the presence of pus cells, epithelial cells, red blood cells and *Candida albicans* positive were estimated under 10 × and 40 × objective lens.

The confirmation of candida infections was performed by observing the colonies from culture for their morphological characteristics such as size, cream-coloured pastry colonies, and morphology of the colonies and production of hyphae examined under a 40 × objective lens. Gram stain to identified *Candida sp* and staining with lactophenol cotton blue, *Candida sp* were finally examined under 40 × and 100 × under oil emersion [20].

### Statistical analysis

The results of this study were presented in the form of tables and figures. Frequencies and percentages where appropriate were used to present the results of relevant variables. The Clopper-Pearson test was used to determine the confidence intervals of proportions of relevant outcome variables under study. The relative risk and its associated confidence intervals were used to assess the risk of candidiasis stratified by sociodemographic and clinical variables. Using the 2 × 2 contingency diagnostic test, the diagnostic accuracy of urine and HVS wet mount enumerated RBCs, ECs and PCs were assessed in the diagnosis of candidiasis among the study participants. All p-values less than 0.05 were considered statistically significant. All data analyses were performed using GraphPad vs 9.3.1.

## Results

### Demographic characteristics and relative risk of *Candida* infection among the study subject

The microscopic detection of *Candida* infection at the Outpatient department (OPD) of the University of Cape Coast Hospital showed that 86.4% (740/856) were from high vaginal swabs (HVS) samples and 13.6% (116/856) from urine samples. The high prevalence of candida infection was among female subjects 97.1% (831/856) compared to males 2.9% (25/856). The microscopic profiles which characterized candida infection were pus cells 96.4% (825/856), epithelial cells 98.7% (845/856), red blood cells (RBCs) 7.6% (65/856) and *Candida albicans* positive 63.2% (541/856). There was high *Candida* infection among the age category 20–29 years old was 61.9% (525/848), followed by 30–39 years old 19.3% (164/848). The highest candidiasis infection was recorded in 2017 22.3% (191/856), followed by 2019 20.2% (173/856) and 2014 15.5% (133/856) (Table 1).

**Table 1:** Characteristics and relative risk of candidiasis infections

Characteristics	n	% (95% CI%)	RR (95% CI)
Sample			
Urine	116	13.6 (11.21–15.86)	0.271 (0.227–0.322)
High vagina swab	740	86.4 (83.03–87.83)	
Microscopy (Wet mount)			
Pus cells	825	96.4 (93.76–96.68)	1.908 (1.817–2.006)
Epithelial cells	845	98.7 (96.45–98.58)	1.954 (1.864–2.052)
Red blood cells	65	7.6 (5.85–9.48)	0.150 (0.118–0.190)
Yeast cells	541	63.2 (59.22–65.78)	1.251 (1.166–1.341)
Sex			
Male	25	2.9 (1.97–4.37)	0.061 (0.041–0.088)
Female	831	97.1 (94.55–97.26)	
Age categories/year^a^			
1–19	95	11.2 (8.98–13.26)	0.219 (0.180–0.266)
20–29	525	61.9 (57.35–63.97)	1.214 (1.129–1.303)
30–39	164	19.3 (16.4–21.73)	0.379 (0.327–0.438)
40–49	49	5.8 (4.22–7.42)	0.113 (0.086–0.149)
50–59	6	0.7 (0.25–1.5)	0.015 (0.007–0.032)
60–69	9	1.1 (0.48–1.97)	0.022 (0.011–0.041)
Yearly pattern			
2013	46	5.4 (3.92–7.03)	0.106 (0.079–0.141)
2014	133	15.5 (13.04–17.95)	0.308 (0.261–0.361)
2015	95	11.1 (8.98–13.26)	0.219 (0.180–0.266)
2016	96	11.2 (9.08–13.38)	0.222 (0.182–0.269)
2017	191	22.3 (20.35–26.08)	0.462 (0.405–0.526)
2018	95	11.1 (8.98–13.26)	0.219 (0.180–0.266)
2019	173	20.2 (17.38–22.82)	0.409 (0.355–0.469)
2020	27	3.2 (2.07–4.51)	0.062 (0.043–0.090)

^a^Indicates 10 missing age data

The relative risk (RR) of detecting *Candida* infection in HVS compared with urine samples was significantly high RR (95% CI), 0.271 (0.227–0.322). The relative risk of detecting pus cells, epithelial cells, RBCs and *Candida albicans* positive at high power fields light microscopy were RR (95% CI), 1.908 (1.817–2.006), 1.954 (1.864–2.052), 0.150 (0.118–0.190) and 1.251 (1.166–1.341) respectively.

### Microscopic profile of candida infection and the severity of infections of candidiasis

The prevalence of candidiasis above > 15ECs/HPF, > 5RBCs/HPF and > 5ECs/HPF were previously considered severe candidiasis in wet mounts [21, 22]. The cut-off point for microscopic detection of *Candida* infection profile at a high-power field was classified as > 5 RBCs/HPF, > 15 epithelial cells (ECs)/HPF and > 5 pus cells (PCs)/HPF were assessed across the eight years (from 2013 to 2020). There was low detection of RBCs in candida infection across the eight years with 2015, 2016, 2013 and 2014 having a slightly high prevalence of 10.9% (10/91), 10.4% (10/96), 8.7% (4/46) and 8.3% (11/133) respectively. The epithelial cells profile in candidiasis were relatively high compared to RBCs and pus cells with 2020, 2015, 2018, 2016 and 2017 recording 48.1% (13/27), 45.1% (13/27), 43.2 (41/95), 42.7% (41/96) and 42.4% (81/191) respectively. A high pus cell profile of 25.3% (24/95), 23.1% (21/91), 22.2% (6/27) and 23.0% (44/191) were recorded in 2018, 2015, 2020 and 2017 respectively (Table 2).

**Table 2:** Microscopic profile of candidiasis and the severity of infection

	> 5 RBCs/HFP	> 15EC/HPF	> 5PC/HPF	RBCs/ECs		RBCs/PCs	
Year	n/N (%)	n/N (%)	n/N (%)	X2	p	X2	P
2013	4/46 (8.7)	14/46 (30.4)	8/46 (17.4)	4.685	0.030	1.181	0.277
2014	11/133 (8.3)	11/133 (8.3)	20/133 (15.0)	0	> 0.999	2.342	0.126
2015	10/91 (10.9)	41/91 (45.1)	21/91 (23.1)	14.98	< 0.001	3.344	0.067
2016	10/96 (10.4)	41/96 (42.7)	18/96 (18.8)	15.13	< 0.001	1.997	0.158
2017	13/190 (6.8)	81/191 (42.4)	44/191 (23.0)	40.6	< 0.001	14.83	0.001
2018	3/95 (3.2)	41/95 (43.2)	24/95 (25.3)	27.37	< 0.001	14.44	0.0001
2019	12/173 (6.9)	55/173 (31.8)	33/173 (19.1)	23.37	< 0.001	8.697	0.003
2020	1/27 (3.7)	13/27 (48.1)	6/27 (22.22)	8.431	0.004	3.183	0.074

The yearly severity of Candidiasis was determined by the yearly ratio of RBCs/ECs and RBCs/PCs compared with the overall RBCs/ECs ratio and RBCs/PCs ratio. The chi-square analysis showed highly significant association of RBCs/ECs ratio in severe candida infection for 2015 (χ2 = 14.98, *p* < 0.001), 2016 (χ2 = 15.13, *p* < 0.001), 2017 (χ2 = 40.6, *p* < 0.0001), 2018 (χ2 = 27.37, *p* < 0.001), 2019 (χ2 = 23.37, *p* < 0.001) and 2020 (χ2 = 8.431, *p* < 0.001). However, RBCs/PCs ratio showed significant difference in 2017 (χ2 = 14.83, *p* < 0.001), 2018 (χ2 = 14.14, *p* < 0.001) and 2019 (χ2 = 8.697, *p* = 0.003) (Table 2).

### Distribution of *Candida* infection profiles across age categories and the year of diagnosis

**Fig. 3:**
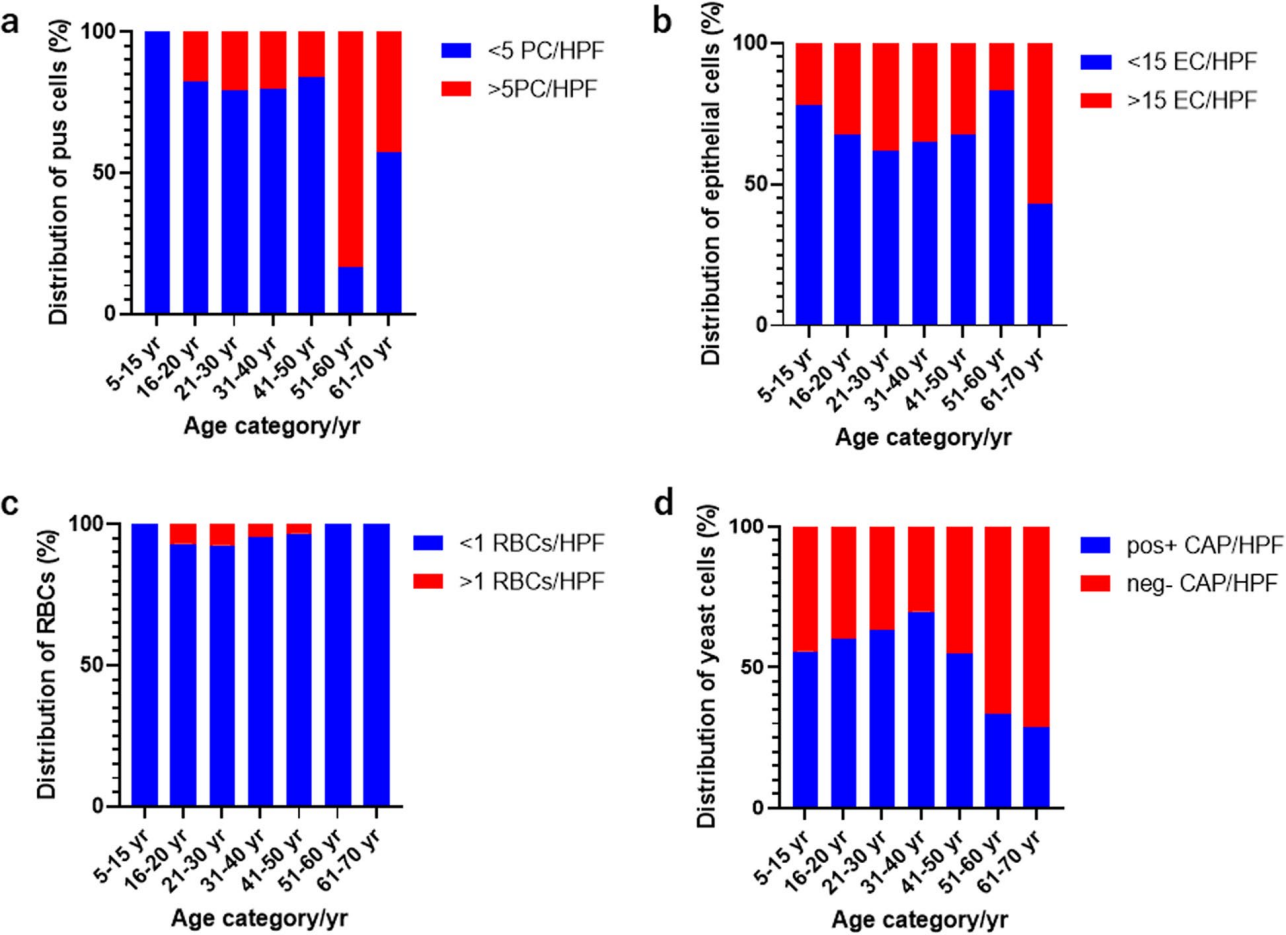
Distribution of candidiasis profile across the stratified age categories among wet mount preparation with cultured diagnosis candidiasis. **a** The distribution of pus cells per high power field across the stratified age categories among wet mount preparation. **b** The distribution of epithelial cells per high power field across the stratified age categories among wet mount preparation. **c** The distribution of red blood cells per high power field across the stratified age categories among wet mount preparation. **d** The distribution of yeast cells (*Candida albicans*) positive per high power field across the stratified age categories among wet mount preparation

**Fig. 4:**
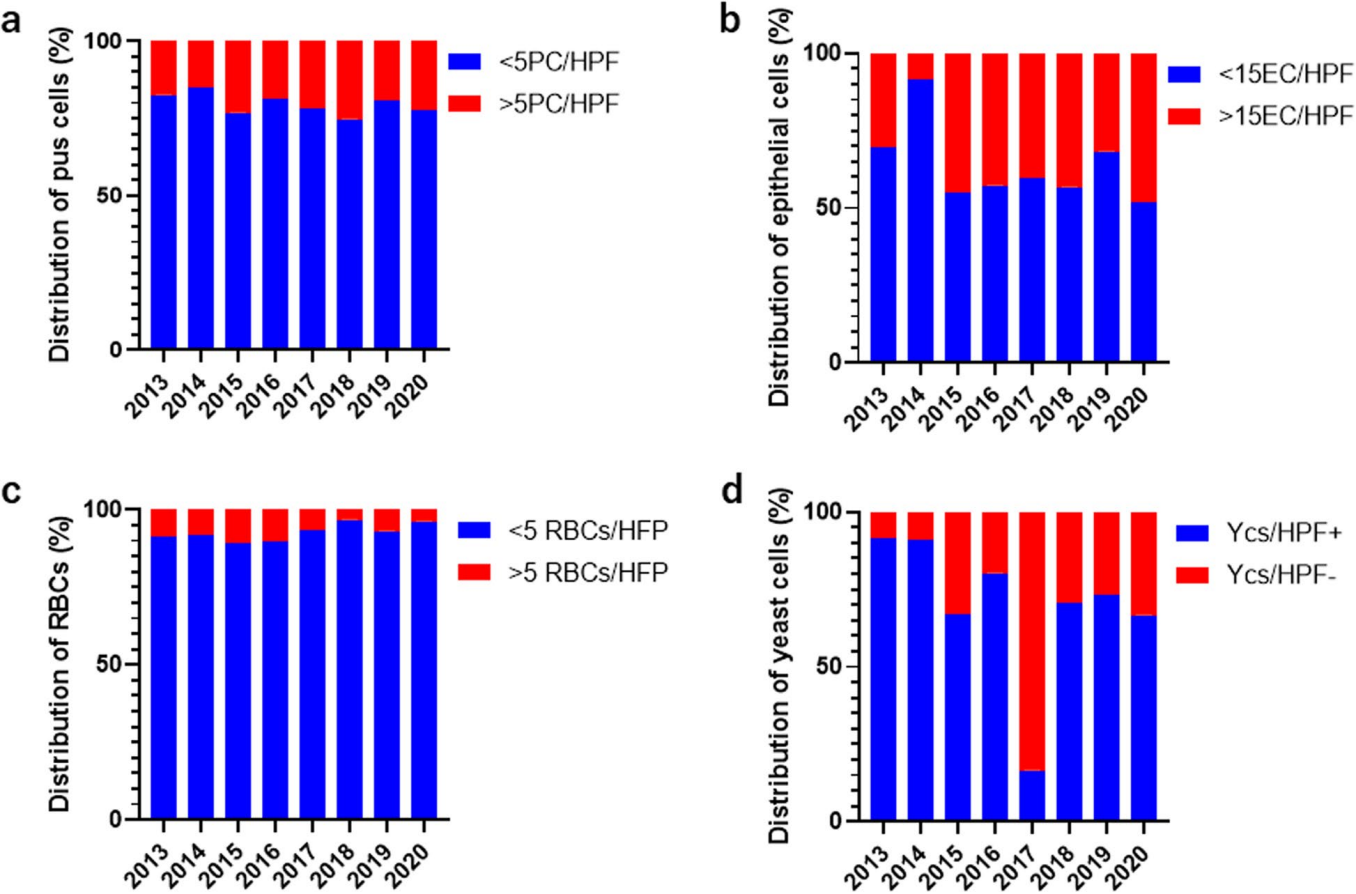
Distribution of candidiasis profile across the year of diagnosis among wet mount preparation with cultured diagnosis candidiasis. **a** The distribution of pus cells per high power field across the year of diagnosis among wet mount preparation. **b** The distribution of epithelial cells per high power field across the year of diagnosis among wet mount preparation. **c** The distribution of red blood cells per high power field across the year of diagnosis among wet mount preparation. **d** The distribution of yeast cells (*Candida albicans*) positive per high power field across the year of diagnosis among wet mount preparation

Candidiasis was more prevalent among the age group 21–30 years 57% (482/845) followed by 16–20 years 18.7% (158/845) and 31–40 years 18% (152/845) (Table 3). The detection and classification of *Candida* infection profiles (presence of pus cells (PCs), epithelial cells (ECs), red blood cells (RBCs), and *Candida albicans* positive) at a high-power field (HPF) under a light microscope showed that majority of the candidiasis infections had lower than 5 PC/HPF except at the age group 51–60 years which had 83.3% (3/6) and 61–70 years 42.9% (3/7). The prevalence of more than 15 EC/HPF ranges from 16.7% (1/6) in the age group 51–60 years to 57.1% (4/7) in the age group 61–70 years. The prevalence of more than 15 ECs/HPF were 38.2% (184/482), 34.9% (53/152) and 32.3% (51/158) among the age groups 21–30 years, 31–40 years and 16–20 years respectively. The detection of more than 5 RBCs/HPF among candidiasis infection ranges from 3.2% (1/31) among the age group 41–50 years to 7.5% (36/482) among 21–30 years. The microscopic detection of *Candida albicans* positive in urine or HVS samples under light microscopy ranged from 28.6% (2/7) among aged 61–70 years to 69.7% (106/152) among 31–40 years (Table 3, Fig. 3). A similar pattern of *Candida* infection profile was observed across the year of diagnosis (Fig. 4).

**Table 3:** Prevalence of candidiasis profiles among the age categories

		PC/HPF, n/N (%)		EC/HPF, n/N (%)		RBCs/HPF, n/N (%)		CAP^a^/HPF, n/N (%)	
Age/yr	Prevalence n/N (%)	< 5	> 5	< 15	> 15	< 5	> 5	yes	no
5–15	9/845 (1.1)	9/9 (100)	-	7/9 (77.8)	2/9 (22.2)	9/9 (100)	-	5/9 (55.6)	4/9 (44.4)
16–20	158/845 (18.7)	130/158 (82.3)	28/158 (17.7)	107/158 (67.7)	51/158 (32.3)	147/158 (93.0)	11/158 (7.0)	95/158 (60.1)	63/158 (39.9)
21–30	482/845 (57.0)	381/482 (79.0)	101/482 (21.0)	298/482 (61.8)	184/482 (38.2)	446/482 (92.5)	36/482 (7.5)	304/482 (63.1)	178/482 (36.9)
31–40	152/845 (18.0)	121/152 (79.6)	31/152 (20.4)	99/152 (65.1)	53/152 (34.9)	145/152 (95.4)	7/152 (4.6)	106/152 (69.7)	46/152 (30.3)
41–50	31/845 (3.7)	26/31 (83.9)	5/31 (16.1)	21/31 (67.7)	10/31 (32.3)	30/31 (96.8)	1/31 (3.2)	17/31 (54.8)	14/31 (45.2)
51–60	6/845 (0.7)	1/6 (16.7)	5/6 (83.3)	5/6 (83.3)	1/6 (16.7)	6/6 (100)	-	2/6 (33.3)	4/6 (66.7)
61–70	7/845 (0.8)	4/7 (57.1)	3/7 (42.9)	3/7 (42.9)	4/7 (57.1)	7/7 (100)	-	2/7 (28.6)	5/7 (71.4)

CAP^a^ *Candida albicans* positive

### The sensitivity and specificity of *Candida* infection profiles for microscopic diagnosis of severe candidiasis

The potential of the individual *Candida* infection profiles to diagnose severe candidiasis were tested for their sensitivity and specificity. The sensitivity (95%) for detecting *Candida albicans* positive and red blood cells (0.62 (0.59–0.65)), *Candida albicans* positive and pus cells (0.75 (0.72–0.78)) and *Candida albicans* positive and epithelial cells (0.95 (0.92–0.96)) with corresponding specificity (95% CI) of 0.63 (0.60–0.67), 0.69 (0.66–0.72) and 0.74 (0.71–0.76) were detected among the high vaginal swab samples (Table 4). The sensitivity of detecting *Candida albicans* positive and red blood cells (0.83 (0.73–0.90)), *Candida albicans* positive and pus cells (0.78 (0.67–0.86)) and *Candida albicans* positive and epithelial cells (0.98 (0.91–0.99)) with corresponding specificity (95% CI) of 0.65 (0.57–0.72), 0.66 (0.58–0.73) and 0.67 (0.59–0.74) were detected among the urine samples (Table 5).

**Table 4:** The pattern of candidiasis profiles and their comparative diagnostic sensitivity and specificity in HVS samples

Characteristics/HPF	Sensitivity (95% CI)	Specificity (95% CI)	PPV (95% CI)	NPV (95% CI)
*Candida albicans* positive vs Epithelial cells	0.62 (0.59–0.65)	0.63 (0.60–0.67)	0.65 (0.62–0.69)	0.60 (0.56–0.63)
*Candida albicans* positive vs Pus cells	0.75 (0.72–0.78)	0.69 (0.66–0.72)	0.65 (0.62–0.68)	0.79 (0.75–0.81)
*Candida albicans* positive vs RBCs	0.95 (0.92–0.96)	0.74 (0.71–0.76)	0.65 (0.62–0.69)	0.96 (0.95–0.97)

**Table 5:** The pattern of candidiasis profiles and their comparative diagnostic sensitivity and specificity in urine samples

Characteristics/HPF	Sensitivity (95% CI)	Specificity (95% CI)	PPV (95% CI)	NPV (95% CI)
*Candida albicans* positive vs Epithelial cells	0.83 (0.73–0.90)	0.65 (0.57–0.72)	0.51 (0.42–0.60)	0.90 (0.83–0.94)
*Candida albicans* positive vs Pus cells	0.78 (0.67–0.86)	0.66 (0.58–0.73)	0.51 (0.42–0.60)	0.86 (0.79–0.91)
*Candida albicans* positive vs RBCs	0.98 (0.91–0.99)	0.67 (0.59–0.74)	0.51 (0.42–0.60)	0.99 (0.95–0.99)

## Discussion

The early diagnosis of candidiasis and prompt treatment is critical for better-quality management outcomes [2, 16, 23]. However, vulvovaginal candidiasis has not received the attention it deserves. The standards of care for vulvovaginal candidiasis are not well defined [8, 24], especially in developing countries like Ghana. The methods of clinical diagnosis of candidiasis based on the clinical symptoms and the wet mount preparation lack sensitivity [13, 15]. However, they remain the diagnostic method for candidiasis, especially in most healthcare facilities in Ghana. The tremendous effort to report incidences, the risk factors and pathogenic mechanisms underlying *Candida* infections could affect the diagnosis of candidiasis [7, 25]. This study assessed the sensitivity and specificity of ECs, PCs, and RBCs as candidiasis profiles in wet mount preparation of urine and HVS samples and their diagnostic potential.

The prevalence of candidiasis was high among 20 to 29 years old, followed by 30 to 39 years old. A study has reported a similar prevalence among pregnant women from Ibadan in Nigeria [26]. Another study reported a higher prevalence of *Candida species* among those aged 30 to 36 years [27]. Although Candida infections are well known to affect women at their reproductive age, the high prevalence within these age groups [10, 28] is not well understood. However, the use of contraceptives and antibiotics have been shown as risk factors for candida infection among these age groups [29, 30].

The vaginal ECs shedding and secretion of mucin and interepithelial cell connections impairs Candida invasion [31]. The presence of vaginal ECs decreases Candida infections in women with diagnosed recurrent VVC having a reduced anti-Candida activity by ECs [31, 32]. The eight years of observation in this study augment the reduction of vaginal ECs in candidiasis. The ECs suggests a protective role against Candida species [31]. This cardinal profile of ECs makes it an essential characteristic factor for diagnosis. The sensitivity of ECs as a *Candida* profile for diagnosis was 0.34 for the diagnosis of candidiasis. The results suggest a range of innate immune mechanisms against Candida infections.

The study revealed that most subjects had < 5 pus cells/HPF across the eight years. Other studies could not establish high PCs in funguria and VVC cases [33]. The low PCs in candidiasis and increased expression of TLR4 is associated with crosstalk between polymorphonuclear cells (mainly neutrophils) and ECs [33]. Thus, the PCs and ECs in candidiasis is under the control of the inflammatory response [31, 34]. The sensitivity of PCs as candidiasis profile was 0.23 which is consistent with previous observation [33, 34].

Rarely has RBCs in urine or HVS been classified as a symptom of candidiasis [35–37]. However, the study observed a small subgroup of Candida infections with blood in their samples. The RBCs could suggest an active invasion of *Candida sp* or the destruction of the vaginal protective ECs. In the invasive hyphal form, the secretion of aspartyl proteases and candidalysin could cause tissues damage [38, 39]. The Candida burden could trigger an intense inflammatory response [32, 38]. The results showed a significant association between the severity of candidiasis and the ratio of RBCs and ECs. The diagnostic accuracy of RBCs, epithelial, and pus cells was high VVC diagnosis in both urine and HVS samples. The low positive predictive values especially among the urine samples suggest that a large proportion of patients with VVC will inevitably be diagnosed as negative and therefore will require highly sensitive detection methods such as culture and molecular diagnosis. Also, the ratio of RBCs/ECs or RBCs/PCs showed a significant association with severe candidiasis compared to RBCs. Thus, a higher ratio of RBCs/ECs or RBCs/PCs together with clinical symptoms of candidiasis and positive Candida albicans in a wet mount preparation could suggest severe or invasive candidiasis.

In conclusion, the study has shown that the presence of PCs, ECs, RBCs or ratio of RBCs/ECs and RBCs/PCs in the wet mount preparation from urine or HVS can enhance microscopic diagnosis of VVC cases. The RBCs have higher specificity and sensitivity but low positive predictive values indicating that many VVC patients may not have RBCs in their urine or HVS samples which could lead to misdiagnosis. Combining the clinical symptoms with candidiasis profiles in wet mount preparation without *Candida albicans* positive requires sensitive diagnostic methods such as culture for confirmation and prompt treatment.

## Data Availability

All data generated or analysed during this study are included in this published article.
